# Acute Poisonings Admitted to the Intensive Care Unit of Cocody University Hospital, Côte d’Ivoire, Africa: Clinical Characteristics and Factors Associated With Intensive Care Unit Mortality

**DOI:** 10.7759/cureus.116812

**Published:** 2026-09-23

**Authors:** Setondji Emmanuel Raymond Ahouangansi, Floriane Mouafo, Loes Koffi, Kouame Koffi Isidore, Walamitien Cyrille Touré, Yao Vianney Bedie, Etienne Spah N'Dah, Kadidja Koné, Stephane Charles Evrard Adingra, Herna Moche

**Affiliations:** 1 Anesthesia and Critical Care, Université Félix Houphouët-Boigny, Abidjan, CIV; 2 Intensive Care, Yopougon University Hospital, Abidjan, CIV; 3 Anesthesia, Intensive Care, and Emergency Medicine, Yopougon University Hospital, Abidjan, CIV; 4 Intensive Care, Treichville University Hospital, Abidjan, CIV

**Keywords:** acute poisoning, côte d'ivoire, emergency department, intensive care, prognosis, triage

## Abstract

Background: Acute poisoning is a common reason for emergency department (ED) visits and intensive care unit (ICU) admission in sub-Saharan Africa. This study aimed to describe the clinical and toxicological characteristics of acute poisonings admitted to the ICU and to identify clinical and circumstantial factors associated with ICU mortality in Côte d’Ivoire.

Methods: This is a retrospective, observational, single-center study conducted from January 2020 to December 2022. All patients admitted to the ICU for acute poisoning were included. Sociodemographic, clinical, toxicological, and emergency management data were analyzed using Microsoft Excel 2016 (Microsoft Corp., Redmond, WA) and Epi Info 7.2 (CDC, Atlanta, GA). Chi-square or Fisher's exact tests were used (significance threshold: p < 0.05).

Results: Of 944 ICU admissions, 296 (31.4%) were for acute poisoning; 200 were analyzed after excluding incomplete records. All patients were initially assessed in the hospital emergency department before ICU transfer. The mean age was 17.4 ± 15.1 years; 53% were female; 48% were under 15 years. Voluntary ingestion accounted for 55% of cases. Caustic substances (48%), organophosphates (17%), and medications (14%) were the most common agents. The overall ICU mortality was 16%. Factors significantly associated with ICU death included age under 15 years (p = 0.005), petroleum product exposure (odds ratio (OR): 3.90 (1.37-11.10); p = 0.005), seizures (OR: 12.06 (5.37-27.26); p < 0.001), respiratory distress (OR: 2.37 (1.18-4.77); p = 0.043), cardiovascular collapse (OR: 6.09 (3.27-10.96); p < 0.001), and dehydration (OR: 26.35 (8.26-106.33); p < 0.001). Additional factors associated with death included delayed ED presentation (≥24 hours: OR: 14.40 (2.43-85.33); p = 0.004) and prehospital oil-based emetic use (OR: 2.08 (1.02-4.24); p = 0.04).

Conclusion: Acute poisoning predominantly affects children and young adults. Early triage in the ED, recognition of severity signs, and prompt referral are critical to improving outcomes. These findings have direct implications for emergency medicine practice in resource-limited settings.

## Introduction

Acute poisoning refers to a set of non-therapeutic pathological phenomena occurring after the inhalation, ingestion, injection, or absorption of a toxic substance within less than 24 hours, leading to disruption of the body's homeostasis and temporary or complete impairment of vital functions [1]. According to the World Health Organization (WHO), it includes cellular and tissue injuries, functional disorders, or even death caused by exposure to toxic substances [2].

Acute poisoning affects both children and adults and represents a major concern for prehospital medicine, emergency departments, and intensive care units (ICUs) in many countries worldwide [3]. The hospital prevalence of acute poisoning varies depending on regions and age groups. In Quebec, for example, emergency departments reported 7,055 cases of acute alcohol poisoning among individuals aged 12 years and older over an 11-month period in 2017, corresponding to a crude rate of 96.18 visits per 100,000 people [4]. In Côte d’Ivoire, the hospital prevalence of acute poisoning in intensive care units has been estimated at 8.8%, with a predominance among pediatric patients [3]. Due to their frequency, etiological diversity, and potential severity, acute poisonings constitute a major global public health problem and are the focus of increasing clinical research. In children, they are often benign despite their high frequency, although some may lead to severe complications or death. In adults, however, the clinical presentation is highly variable, ranging from mild forms to multiorgan failure with potentially fatal outcomes, as highlighted by the WHO in 2013 [3]. Easy access to toxic substances, especially in domestic or occupational settings, plays a significant role in the increasing number of cases among adults. In developed countries such as France, the establishment of poison control centers has allowed for more specialized and coordinated management of acute poisonings. In contrast, in Africa, such structures are almost nonexistent, leaving intensive care units as the primary providers of care for these patients [5].

On March 11, 2020, the World Health Organization (WHO) declared the COVID-19 pandemic, leading to the implementation of strict health measures in several countries, including Canada: lockdowns, curfews, and closures of schools and non-essential businesses. Although overall pediatric emergency visits declined, the proportion of consultations for injuries, poisonings, and mental health disorders increased, and poison control centers reported a rise in calls during the first two months of the pandemic [6]. Following this health crisis, few studies have addressed acute poisonings in intensive care in Africa, particularly in Côte d’Ivoire. The present study aimed to describe the clinical characteristics and prognostic factors of acute poisonings admitted to the intensive care unit of Cocody University Hospital.

## Materials and methods

Study design and setting

This was a retrospective, observational, single-center study with descriptive and analytical objectives, conducted over 36 months (January 1, 2020 to December 31, 2022) in the Intensive Care Unit of Cocody University Hospital (Centre Hospitalier Universitaire (CHU) de Cocody), Abidjan, Côte d'Ivoire. Cocody University Hospital is a tertiary referral center serving a catchment population of approximately one million residents in Abidjan. The ICU is a 12-bed mixed medical-surgical unit. The hospital emergency department (ED), located in the same building, is the primary port of entry for all acute medical presentations, including poisonings. The ED operates 24 hours a day, 7 days a week, and employs a physician-led triage system using clinical assessment (consciousness level, respiratory status, hemodynamic stability, and toxicological history). Patients deemed to require intensive care, based on the presence of coma, respiratory failure, hemodynamic instability, refractory seizures, or high-risk toxic exposure, are transferred to the ICU directly from the ED, typically within one hour of initial assessment.

Study population

All patients of both sexes and all ages admitted to the ICU for acute poisoning during the study period were eligible for inclusion. Acute poisoning was defined as any exposure to a toxic substance (ingested, inhaled, injected, or absorbed) occurring within the 24 hours preceding presentation, with clinical signs attributable to that substance.

Exclusion criteria were as follows: (1) foodborne infectious illness (e.g., salmonella and botulism), (2) subacute or chronic intoxication, (3) envenomation by animal venom (snake and scorpion), and (4) incomplete medical records with missing key variables (outcome, toxic agent, or clinical presentation). Records with more than 30% missing data were excluded.

Data collection

Data were extracted from medical records, nursing notes, and ED admission registers. The following variables were collected: (a) sociodemographic (age, sex, occupation, and level of education), (b) emergency and prehospital data (time from toxic exposure to ED presentation, prehospital first aid measures (including traditional emetic practices such as oil or milk ingestion), and referral source (self-referral, community health center, or other hospital)), (c) clinical data at ED presentation and upon ICU admission (vital signs, Glasgow Coma Scale (GCS) or Blantyre score (for children), clinical signs (seizures, respiratory distress, cardiovascular collapse, miosis, and dehydration)), (d) toxicological data (nature of the toxic agent, route of exposure, and circumstances of poisoning (intentional versus accidental)), (e) ICU management (type of treatments administered and length of ICU stay), (f) outcome (survival or death before discharge).

Statistical analysis

Quantitative variables were expressed as mean ± standard deviation (SD) when normally distributed, or as median (interquartile range) otherwise. Qualitative variables were expressed as frequencies and percentages. Comparisons used the chi-square test for qualitative variables, or Fisher's exact test when expected frequencies were below 5. Odds ratios (OR) with 95% confidence intervals (CI) were calculated for factors significantly associated with mortality. A p-value < 0.05 was considered statistically significant. All analyses were performed using Microsoft Excel 2016 (Microsoft Corp., Redmond, WA) and Epi Info 7.2 (CDC, Atlanta, GA).

Ethics

The study was conducted in accordance with the ethical principles of the Declaration of Helsinki. Ethical approval was obtained from the Institutional Ethics Committee of Cocody University Hospital, Abidjan, Côte d'Ivoire (reference available upon request). Given the retrospective, observational nature of the study and the use of fully anonymized data, the requirement for individual informed consent was waived by the ethics committee. No patient-identifying information was retained in the study database.

## Results

During the study period, 944 patients were admitted to the ICU, including 296 for acute poisoning, representing a prevalence of 31.36%. After excluding 96 incomplete or missing records, 200 patients were included in the analysis. The incidence of acute poisoning was highest in 2020, with a statistically significant difference compared to 2021 and 2022 (chi-square test, p = 0.024).

The population was predominantly female (53%), corresponding to a male/female sex ratio of 0.89 (Table 1). The mean age was 17.4 ± 15.1 years (range: 2 months to 83 years). Nearly half (42%) were children aged 0-14 years, followed by young adults aged 15-29 years (40%) and adults aged 30-45 years (14%). The most represented sociodemographic categories were preschool children (38%), students (18%), and informal sector workers (16%). Regarding past medical history, 5% of patients were drug users, and 3% had a history of suicide attempts. Poisonings were intentional in 55% of cases. The delay between symptom onset and ICU admission was less than 6 hours in 61% of cases. The most common toxic agents were caustic substances (48%), followed by organophosphates (17%) and medications (14%) (Table 2). As for emetic practices, 50% of patients had ingested oil, 8% milk, and 4% both, while 38% did not attempt any maneuver. Clinically, most patients were hemodynamically stable; however, 39% presented with tachycardia and 30% with hypotension. The main clinical signs were dehydration (18.5%) and conjunctival pallor (16%). Respiratory distress was observed in 16% of cases, with oxygen desaturation in 34%. Neurologically, 18% of patients had seizures and 13% dizziness. Among adults, 19% had a Glasgow Coma Scale (GCS) score of 13/15 and 4% a score of 9/15. Among children, 18% had a Blantyre score of 3/5, 11% a score of 4/5, and 10% a score of 2/5. The most common symptoms were vomiting (73%) and abdominal pain (35%). The main complications included cardiovascular collapse (40%), hemorrhagic syndrome (6%), and cardiac arrest (3.5%). Anemia was found in 33% of patients. Biologically, most had normal coagulation profiles, but 13% showed hepatic cytolysis. Eight patients had metabolic acidosis, while 40% had a normal electrolyte panel. Radiologically, the most frequent findings were bilateral alveolo-interstitial opacities (19 cases). Endoscopic examination revealed edema or erythema (stage 1) in 9% of patients. Management mainly relied on oxygen therapy (42%), fluid resuscitation (54%), and electrolyte administration (47%). The overall case fatality rate was 18.5%, with a mean hospital stay of 1.5 days.

**Table 1:** Correlation Between Outcome and Age Group CI: confidence interval, OR: odds ratio

Age group	Survivors (n = 163)	Deceased (n = 37)	p-value	OR (95% CI)	Statistical test
0-14 years	59	25	0.005	2.40 (1.02-6.84)	Chi-square test
15-29 years	72	8	0.008	3.45 (1.40-8.48)	Chi-square test
30-45 years	25	3	0.192	0.49 (0.15-1.71)	Chi-square test
>45 years	7	1	0.546	0.62 (0.07-5.93)	Fisher's exact test

**Table 2:** Correlation Between Outcome, Toxic Agent, and Emetic Measures CI: confidence interval, OR: odds ratio

Parameter	Survivors (n = 163)	Deceased (n = 37)	p-value	OR (95% CI)	Statistical test
Emetic measures					
None	66	10	0.12	0.55 (0.25-1.18)	Chi-square test
Oil ingestion	78	22	0.04	2.08 (1.02-4.24)	Chi-square test
Oil and milk	5	4	0.07	3.65 (0.87-15.31)	Fisher's exact test
Milk only	14	1	0.26	0.31 (0.04-2.43)	Fisher's exact test
Toxic agent					
Caustic substances	82	14	0.20	0.62 (0.3-1.28)	Chi-square test
Organophosphates	28	5	0.61	0.77 (0.28-2.13)	Chi-square test
Medications	24	4	0.53	0.70 (0.23-2.13)	Chi-square test
Kerosene/petroleum	12	8	0.005	4.02 (1.51-10.69)	Fisher's exact test
Unidentified	3	4	0.01	6.47 (1.62-39.52)	Fisher's exact test
Alcohol	8	1	0.58	0.55 (0.07-4.49)	Fisher's exact test
Rodenticides	2	1	0.50	2.30 (0.20-26.41)	Fisher's exact test

Statistical analysis identified several factors associated with ICU mortality. Age was significantly associated with death: twofold higher in the 0-14 years group (OR: 2.40 (1.02-6.84); chi-square test, p = 0.005) and threefold higher in the 15-29 years group (OR: 3.45 (1.40-8.48); chi-square test, p = 0.008) (Table 1). Regarding toxic agents, mortality was significantly higher with petroleum product ingestion (OR: 4.02 (1.51-10.69); Fisher's exact test, p = 0.005) and unidentified toxins (OR: 6.47 (1.62-39.52); Fisher's exact test, p = 0.01) (Table 2). Prehospital oil ingestion was significantly associated with death (OR: 2.08 (1.02-4.24); chi-square test, p = 0.04) (Table 2). Regarding admission delay, arrival within 6 hours was associated with lower mortality (OR: 0.22 (0.10-0.50); chi-square test, p = 0.0002), while a delay of 6-12 hours was associated with a 3-fold increase (OR: 3.19 (1.50-6.58); chi-square test, p = 0.002), a delay of 12-23 hours with a 6-fold increase (OR: 5.76 (1.35-24.56); Fisher's exact test, p = 0.02), and a delay of 24 hours or more with a 14-fold increase (OR: 14.40 (2.43-85.33); Fisher's exact test, p = 0.004) (Table 3). Several clinical signs at ICU admission were significantly associated with death: dehydration (OR: 26.4 (8.26-106.3); chi-square test, p < 0.0001), seizures (OR: 12.1 (5.37-27.3); chi-square test, p < 0.0001), cardiovascular collapse (OR: 6.09 (3.27-10.96); chi-square test, p < 0.0001), cardiac arrest (OR: 6.45 (1.23-34.3); Fisher's exact test, p = 0.007), respiratory distress (OR: 2.37 (1.18-4.77); chi-square test, p = 0.043), and miosis (OR: 2.43 (1.20-4.96); chi-square test, p = 0.016) (Table 4). Certain therapeutic interventions were associated with higher mortality, with odds ratios ranging from 3.2 to 14.0; these associations reflect confounding by indication rather than treatment harm (Table 5). Finally, a length of ICU stay of 4-6 days was associated with an 11-fold increase in mortality compared to the reference group of 1-3 days (OR: 11.09 (2.72-44.95); Fisher's exact test, p = 0.001), while a stay of less than 1 day was not significantly associated with death (OR: 1.85 (0.36-9.52); Fisher's exact test, p = 0.480) (Table 6).

**Table 3:** Correlation Between Outcome and Time From Symptom Onset to ED/ICU Admission CI: confidence interval, ED: emergency department, ICU: intensive care unit, OR: odds ratio

Time interval	Survivors (n = 163)	Deceased (n = 37)	p-value	OR (95% CI)	Statistical test
<6 hours	103	10	0.0002	0.22 (0.10-0.50)	Chi-square test
6-12 hours	43	19	0.002	3.19 (1.5-6.58)	Chi-square test
12-23 hours	5	4	0.02	5.76 (1.35-24.56)	Fisher's exact test
≥24 hours	2	4	0.004	14.40 (2.43-85.33)	Fisher's exact test

**Table 4:** Correlation Between Outcome and Clinical Signs CI: confidence interval, OR: odds ratio

Clinical sign	Survivors (n = 163)	Deceased (n = 37)	p-value	OR (95% CI)	Statistical test
Dehydration	12	25	0.0001	26.4 (8.26-106.3)	Chi-square test
Seizures	16	19	0.0001	12.1 (5.37-27.3)	Chi-square test
Cardiovascular collapse	51	28	0.0001	6.09 (3.27-10.96)	Chi-square test
Cardiac arrest	3	4	0.007	6.45 (1.23-34.3)	Fisher's exact test
Respiratory distress	22	10	0.043	2.37 (1.18-4.77)	Chi-square test
Miosis	22	11	0.016	2.43 (1.20-4.96)	Chi-square test

**Table 5:** Correlation Between Outcome and Treatment Received CI: confidence interval, OR: odds ratio

Treatment	Survivors (n = 163)	Deceased (n = 37)	p-value	OR (95% CI)	Statistical test
Oxygen therapy	54	30	<0.001	8.65 (3.57-20.96)	Chi-square test
Intubation	14	21	<0.001	13.97 (5.97-32.70)	Chi-square test
Mechanical ventilation	14	21	<0.001	13.97 (5.97-32.70)	Chi-square test
Sedation	17	18	<0.001	8.14 (3.59-18.42)	Chi-square test
Blood transfusion	4	8	<0.001	10.97 (3.10-38.81)	Fisher's exact test
Sodium bicarbonate	4	5	0.003	6.21 (1.58-24.41)	Fisher's exact test
Activated charcoal	15	11	0.001	4.17 (1.73-10.09)	Fisher's exact test
Antibiotic therapy	17	10	0.008	3.18 (1.32-7.69)	Fisher's exact test
Fluid resuscitation	75	32	<0.001	7.51 (2.79-20.24)	Chi-square test
Vasopressors	4	3	0.091	3.51 (0.75-16.39)	Fisher's exact test
Gastric lavage	10	0	0.122	-	Fisher's exact test
Osmotic diuresis	2	1	0.505	2.24 (0.20-25.34)	Fisher's exact test
Dialysis	11	2	0.765	0.79 (0.17-3.72)	Fisher's exact test

**Table 6:** Correlation Between Outcome and Length of ICU Stay CI: confidence interval, ICU: intensive care unit, OR: odds ratio

Length of stay	Survivors (n = 163)	Deceased (n = 37)	p-value	OR (95% CI)	Statistical test
<1 day	7	2	0.480	1.85 (0.20-1.61)	Fisher's exact test
1-3 days	149	23	-	1	Chi-square test
4-6 days	7	11	0.001	11.09 (2.72-44.95)	Fisher's exact test

## Discussion

This study provides a detailed analysis of acute poisonings requiring ICU admission at a tertiary teaching hospital in Abidjan, with particular attention to emergency department referral patterns, clinical severity, and factors associated with ICU mortality. Acute poisoning accounted for 31.4% of ICU admissions during the study period, with a case-fatality rate of 16%. The predominance of young patients, the high proportion of intentional poisonings, the frequent use of potentially harmful prehospital practices, and the association between delayed presentation and mortality highlight several potentially modifiable targets for emergency medicine and public health interventions.

A high burden of acute poisoning in an ICU population

The proportion of acute poisonings observed in our ICU was substantially higher than the 8.8% reported in the intensive care unit of Bouaké, Côte d'Ivoire [3]. This difference may be explained by variations in study populations, referral patterns, ICU admission criteria, and study periods. The high frequency observed in our study may partly reflect the characteristics of Cocody University Hospital as a tertiary referral center. In addition, the development of poison control and toxicovigilance systems has been proposed as an important component of poisoning prevention and management in African countries [5]. The study period also included the COVID-19 pandemic, during which major social and economic disruptions occurred. Previous reports have documented changes in poisoning patterns during periods of social disruption [6]. From an emergency medicine perspective, these findings emphasize the importance of structured initial assessment and early severity stratification in patients presenting with suspected poisoning, particularly in resource-limited settings where access to specialized toxicological services may be limited.

A predominantly young population

The mean age of 17.4 years and the 48% proportion of children younger than 15 years confirm the substantial pediatric burden of acute poisoning in our setting. This observation is consistent with previous studies from Côte d'Ivoire and other African countries [7,8]. Studies conducted in other settings have also demonstrated substantial heterogeneity in the epidemiology of acute poisoning [9]. The predominance of children has important implications for emergency departments because the history of exposure may be incomplete or difficult to obtain, while clinical manifestations may initially be nonspecific. Easy household access to caustic substances and petroleum products may further increase the risk of accidental exposure.

Intentional poisoning and psychosocial assessment

Intentional poisoning accounted for 55% of cases. This finding indicates that acute poisoning is not exclusively an accidental pediatric problem but also represents an important manifestation of intentional self-poisoning, particularly among adolescents and young adults. Intentional poisoning has been described in African populations, including Mali [10], Morocco [11], and Cameroon [12]. These findings support the integration of a psychosocial assessment into the emergency management of patients presenting after intentional poisoning. When clinically appropriate, emergency physicians should consider previous suicide attempts, psychiatric symptoms, substance use, and relevant social circumstances.

Caustic substances as the leading toxic agents

Caustic substances were the most frequently identified toxic agents, accounting for 48% of cases, followed by organophosphates (17%) and medications (14%). This toxicological profile differs from that described in high-income settings, where pharmaceutical agents predominate [13], and is consistent with previous Ivorian series [7,8]. The predominance of caustic substances has direct implications for emergency management. Patients with suspected caustic ingestion require early assessment of airway risk and evaluation of potential gastrointestinal injury. Inducing vomiting should be strictly avoided. In our series, stage 1 mucosal lesions were identified in 9% of patients who underwent endoscopy, likely underestimating the true burden given that endoscopy was not systematically available. Petroleum product ingestion was significantly associated with mortality in bivariate analysis (OR: 4.02 (1.51-10.69); Fisher's exact test, p = 0.005), as was exposure to an unidentified toxic agent (OR: 6.47 (1.62-39.52); Fisher's exact test, p = 0.01). These associations underscore the importance of toxin identification at triage and the need for a systematic toxidrome-based approach when the agent is unknown.

Prehospital emetic practices: a preventable risk factor

One of the most clinically relevant findings was the high frequency of prehospital emetic practices. Half of the patients had ingested oil before hospital arrival, while 8% had received milk and 4% both. Oil ingestion was significantly associated with mortality (OR: 2.08 (1.02-4.24); chi-square test, p = 0.04). Similar practices have been described among children admitted to intensive care in Abidjan [7] and reflect traditional beliefs and limited accessibility to specialized toxicological advice. Families and community healthcare workers should receive clear information regarding appropriate first aid after suspected poisoning and the potential dangers of inducing vomiting or administering unproven substances [5].

Time to emergency department presentation

A delay of 6-12 hours between poisoning and ED presentation was associated with a 3-fold increase in mortality (OR: 3.19 (1.50-6.58); chi-square test, p = 0.002), a delay of 12-23 hours with a 6-fold increase (OR: 5.76 (1.35-24.56); Fisher's exact test, p = 0.02), and a delay of 24 hours or more with a 14-fold increase (OR: 14.40 (2.43-85.33); Fisher's exact test, p = 0.004). Conversely, arrival within 6 hours was associated with lower mortality (OR: 0.22 (0.10-0.50); chi-square test, p = 0.0002). These associations cannot establish causality given the retrospective univariable design, but they highlight the clinical importance of early ED presentation. Delayed presentation may allow progressive toxicity, dehydration, respiratory complications, and cardiovascular instability to develop before definitive treatment. Community education, improved referral networks, and access to toxicological advice could reduce delays between exposure and medical evaluation.

Clinical severity and emergency triage

Several clinical variables were significantly associated with mortality: dehydration (OR: 26.4 (8.26-106.3); chi-square test, p < 0.0001), seizures (OR: 12.1 (5.37-27.3); chi-square test, p < 0.0001), cardiovascular collapse (OR: 6.09 (3.27-10.96); chi-square test, p < 0.0001), cardiac arrest (OR: 6.45 (1.23-34.3); Fisher's exact test, p = 0.007), respiratory distress (OR: 2.37 (1.18-4.77); chi-square test, p = 0.043), and miosis (OR: 2.43 (1.20-4.96); chi-square test, p = 0.016). These findings are consistent with previous reports of severe acute poisoning requiring intensive care [14]. Criminal poisoning has also been documented in North African settings, further illustrating the diversity of circumstances surrounding toxic exposures [15]. The association between aggressive ICU interventions and mortality should be interpreted cautiously. Mechanical ventilation, blood transfusion, activated charcoal, sodium bicarbonate, antibiotics, and fluid resuscitation were more frequently used among patients who died; this reflects confounding by indication rather than treatment-related harm. Altered consciousness, respiratory compromise, seizures, hemodynamic instability, and cardiovascular collapse should prompt immediate stabilization and consideration of early ICU admission.

Mortality compared with other African settings

The overall case-fatality rate of 16% was higher than the mortality reported in Bouaké [3] and consistent with mortality data reported in other African ICU series [16]. It was higher than the 3% reported in Burkina Faso [17] but comparable to the 11%-15% reported in Mali [10,14]. Differences between studies should be interpreted cautiously given variations in case mix, toxic agents, referral delays, and available resources. Our cohort exclusively comprised ICU-admitted patients and therefore represents a selected population with more severe poisoning than the general ED population, which likely contributes to the relatively high case-fatality rate.

Implications for emergency medicine practice

The findings have several practical implications. Emergency departments should implement standardized initial assessment and severity stratification for poisoned patients. Patients with altered consciousness, seizures, respiratory compromise, or hemodynamic instability should receive immediate stabilization and early consideration of ICU admission. Harmful prehospital practices should be addressed through targeted education of families and community healthcare workers. The development of a national toxicological information and toxicovigilance system could facilitate rapid access to expert advice and improve epidemiological surveillance [5]. Prevention strategies should include safe storage and labelling of household and agricultural toxic substances, particularly in homes with young children.

Strengths and limitations

This study provides data from a tertiary ICU in Côte d'Ivoire and describes the pathway from emergency department assessment to ICU admission. However, several limitations must be acknowledged. The retrospective design introduces the possibility of missing or inaccurately recorded data. Exclusion of 96 incomplete records may have introduced selection bias. The single-center design limits generalizability. Because the study was conducted exclusively among ICU-admitted patients, all observed associations between clinical features and mortality pertain to this selected population and cannot be extrapolated to patients managed at ED level. All analyses are univariable; confounding cannot be excluded, and no causal inference is warranted. Future prospective multicenter studies including patients managed at ED level, as well as those subsequently admitted to ICU, would provide a more comprehensive description of the epidemiological and clinical spectrum of acute poisoning in Côte d'Ivoire.

## Conclusions

Acute poisonings accounted for nearly one-third of ICU admissions at Cocody University Hospital, with a case fatality rate of 18.5%. Caustic substances were the most frequently implicated agents, followed by organophosphates and medications. The affected population was predominantly young, and over half of the poisonings were intentional, reflecting the psychosocial impact of the local context. Factors associated with poor outcomes included age ≤ 29 years, delayed admission, emetic practices, seizures, dehydration, cardiovascular collapse, and cardiac arrest. These findings underscore the need to strengthen domestic and community prevention, raise public awareness about the dangers of emetic practices, improve early referral to specialized care, and promote the establishment of a national toxicovigilance center.
